# Prevalence of Psychotic Symptoms and Their Risk Factors in Urban Tanzania

**DOI:** 10.3390/ijerph7062514

**Published:** 2010-06-10

**Authors:** Rachel Jenkins, Joseph Mbatia, Nicola Singleton, Bethany White

**Affiliations:** 1 WHO Collaborating Centre (Mental Health), Institute of Psychiatry, Kings College London, UK; 2 Mental Health, Ministry of Health, Tanzania; E-Mail: jmbatia@muchs.ac.tz; 3 Policy & Research, UK Drug Policy Commission, UK; E-Mail: nicola.singleton@homeoffice.gsi.gov.uk; 4 WHO Collaborating Centre (Mental Health), Institute of Psychiatry, Kings College London, UK; E-Mail: bwhite@nchecr.unsw.edu.au

**Keywords:** Tanzania, psychosis, poverty

## Abstract

This study aimed to determine the prevalence of psychotic symptoms in urban Tanzania and their relationship with demographic, socio-economic and social factors. A random sample of 899 adults aged 15–59 was surveyed. The main outcome measure was endorsement of one or more psychotic symptoms identified by the Psychosis Screening Questionnaire. 3.9% respondents reported one or more psychotic symptoms in the preceding year. Significantly higher rates of symptoms were found in those who had recently experienced two or more stressful life events, those with CMD and people who had used cannabis in the preceding year.

## 1. Introduction

Although psychotic disorders, largely schizophrenia and bipolar disorder, are less common than the non-psychotic disorders such as depression and anxiety, schizophrenia in particular is associated with greater chronic disability than any other mental illness, and the social and economic costs are disproportionately high [1]. Most of the research evidence is based on studies in developed countries with few data available on psychosis in poorer regions, particularly Africa [2] Where surveys of psychosis have been conducted, prevalence rates are broadly similar to those in the developed world [3,4], yet human resources devoted to treatment and care of mental disorders are far less in low income countries [5], especially sub-Saharan Africa [6]. Surveys of psychosis have been conducted using clinician administered instruments [3] which can establish both psychotic symptom severity and diagnostic category; using family reports [4] and using systematic assessment of psychotic symptoms by detailed interviews administered by non-medical interviewers, leading to enumeration of symptom frequency and severity, and estimate of probable psychosis [7,8].

There is considerable research interest in the linkages between health and poverty, and there have now been a number of studies of mental disorders and socio-economic factors in rich countries [9–11], with recent work highlighting the complexity of this relationship [12]. These associations have been investigated to a lesser extent in poor countries [13].

While there has been extensive research on the complex relationship between psychotic symptoms, psychosis and social class in rich countries [7,14,15], there has been less research on the relationship between psychosis and actual poverty. In the UK, people with low incomes are also more likely to be admitted to hospital with psychosis [16] and the first epidemiological study to report the relationships between income, debt and estimates of probable psychosis found that probable psychosis was significantly associated with low income, and with numbers of debts [12].

This paper describes a project which aimed to determine the prevalence of psychotic symptoms in urban Tanzania and their relationship with demographic, socio-economic and social risk factors.

## 2. Methods

### 2.1. Sites

As previously described [17], in September and October 2003 a population-based survey was conducted in two urban areas of Dar es Salaam, Tanzania’s largest city of 2.5 million. The areas were sites of the Adult Morbidity and Mortality Project (AMMP) [18,19], selected to ensure subpopulations of differing socio-economic circumstances. Ilala- Ilala (Ilala municipality) was a relatively middle-income area while Mtoni-Saba Saba (Temeke municipality) was a lower-income area where traders and farmers resided [20].

### 2.2. Sample

The sampling frame used was a database listing the names of all residents in the two areas that had been compiled for the AMMP. Thus it was possible to sample individuals rather than households. A systematic sample of 1,100 adults aged 15–59 was drawn from a random starting point from the previously enumerated populations of two geographically defined areas; 550 from the eligible population of 4,690 in Ilala-Ilala, and 550 from an eligible population of 11,620 in Mtoni-Saba Saba [20]. If the person randomly selected for interview in each household had moved away, the person who had moved into the house was interviewed instead. It was individuals rather than households which were enumerated, formed by the AMMP into a database of names, and hence sampled. Therefore, the sample was designed to be large enough in each area provide estimates with adequate and similar levels of precision. In surveys in which the populations of two different areas are to be compared it is the absolute size of each sample that is important, since this is what determines the precision of estimates, not the proportion of the population that is sampled that is important (unless the sample is getting near to total enumeration of the population).

### 2.3. Procedures

The Mental Health Section of the Ministry of Health, the Health Research Systems Section of the Directorate of Planning, and Dar es Salaam City Health Services coordinated the survey. Interviews were conducted by volunteer community health workers based in primary health care centres, trained in administration of the pencil and paper interview. Written, informed consent was obtained. The instruments were reviewed by local mental health staff for local content validity, translated into Kiswahili and back translated.

### 2.4. Instruments

Demographic characteristics, socio-economic factors, recent life events and perceived social supports were documented. The Psychosis Screening Questionnaire (PSQ) [21], assessed psychotic symptoms, the Clinical Interview Schedule Revised (CIS-R) [22], indicated common mental disorder (Jenkins *et al.*, in preparation), and the Alcohol Use Disorders Identification Test (AUDIT) [19], measured hazardous alcohol use [17].

Demographic information collected included sex, age, marital status, ethnicity and household status (head, spouse or other) were recorded. Socio-economic information documented included employment status, education attainment, income, housing tenure (owned or rented) and type of accommodation (whole house or room only).

The PSQ assessed the past-year presence of psychotic symptoms. The instrument developed for use by lay interviewers employs five probes to determine recent experience of mania, thought insertion, paranoia, strange experience and hallucinations.

The CIS-R [22], is a gold standard instrument for use by lay interviewers in assessing common mental disorders (CMD) in community settings, which has been widely used in low-income countries [23–25], including Tanzania [26]. For the purpose of the current paper, a score of 12 or more across the 14 sections of the survey was considered an indication of any CMD, as used in other CIS-R studies [22].

Respondents were given a list of 18 different stressful life events, and asked to say which, if any, they had experienced in the past six months. The list included relationship problems, employment, financial crises and victimisation experiences. The list was originally developed for the 1993 British psychiatric morbidity survey [27,28], and tailored for the Tanzania context. For the purposes of analysis, life event scores were grouped into “none”, “one” and “two or more” life events.

Perceived social support was assessed from respondents’ answers to seven questions previously used for the 1992 Health Survey for England [29], and the Office of National Statistics (ONS) surveys of psychiatric morbidity [30,31]. Participants responded “true”, “partly true” or “certainly true” in response to the question ‘There are people I know who’; (i) Do things to make me happy; (ii) Who make me feel loved; (iii) Who can be relied on no matter what happens; (iv) Who would see that I am taken care of if I needed to be; (v) Who accept me just as I am; (vi) Who make me feel an important part of their lives; and (vii) Who give me support and encouragement. Results were categorised into no, moderate or severe lack of perceived social support.

Information on social networks was obtained through questions about the number of friends or relatives who informants felt close to including (i) Adults who lived with the respondent and to whom they felt close; (ii) Relatives living elsewhere to whom they felt close; and (iii) Friends or acquaintances living elsewhere who informants would describe as close or good friends. These questions were taken from psychiatric morbidity surveys conducted in Britain [32,33], and results grouped “none to three”, “four to eight” and “nine or more”.

### 2.5. Data Analysis

Data were analysed using SPSS software for Windows Version 15 (SPSS Inc, 2006). Chi squared (χ^2^) tests were conducted to examine demographic and socio-economic differences between the two areas as well as differences in perceived social support and recent life events. The prevalence of each symptom type in the two areas and for the sample overall was calculated. Odd ratios (ORs) with 95% confidence intervals (CIs) were calculated to determine significant associations with the primary outcome variable which was defined as endorsement of at least one psychotic symptom (initial probe and secondary question).

All variables significantly associated with psychotic symptoms as well as factors significantly different between areas were included in the forward stepwise multivariate logistic regression analysis. Where variables were entered into the regression equation in steps. At each step all variables not already included are considered for entry into the equation and the variable that will produce the greatest increment in R-squared is entered into the model. This process is continued until none of the remaining variables make a significant difference to the explanatory power of the model. This method identifies those variables that are independently associated with the outcome variable and the adjusted odds ratios for those variables only. Statistical significance was set at p < 0.05.

### 2.6. Ethics Approval

Approval was granted by National Institute for Medical Research, Ministry of Health, United Republic of Tanzania and South London and Maudsley (SLaM), National Health Service (NHS) Foundation Trust.

## 3. Results

### 3.1. Response Rates

Of the 1,100 households approached, 899 (82%) residents agreed to participate. The frequency of replacement by new residents when the original person selected for interview no longer resided at the household was not recorded.

### 3.2. Demographic, Socio-Economic and Social Differences between Areas

Respondents from Saba Saba and Ilala were of comparable age (34% *vs.* 37% aged 35 years or over, p = 0.51), gender (56% *vs.* 57% male, p = 0.76) and marital status (55% *vs.* 56% married, p = 0.69) but respondents from Ilala were significantly more likely to be household head (35% *vs.* 45%, p < 0.0001), to be non-African ethnicity (2% *vs.* 12%, p < 0.0001) and to report renting their home (48% *vs.* 55%, p = 0.04). Living in poorer Saba Saba was associated with unemployment (9% *vs.* 3% unemployed, p < 0.0001) and younger school leaving age (8% *vs.* 5% left school 13 years or under, p = 0.01) and participants from Saba Saba reported a significantly higher number of life events in the six months preceding interview (7% *vs.* 3% three or more, p < 0.0001).

### 3.3. Prevalence and Risk Factors for Psychotic Symptoms

Thirty five (3.9%) respondents endorsed one or more PSQ item. The annual prevalence of psychotic symptoms was significantly lower in the middle income Ilala compared to the more densely populated Saba Saba (2.1 *vs.* 6.0%, unadjusted OR = 0.33; 95% CI 0.16–0.70, p = 0.004). “Strange experiences” were the most commonly reported symptoms in both areas (Table 1).

Given the small number of people reporting symptoms, the areas were combined when examining factors correlated with symptoms, nevertheless, the overall small number of cases made it difficult to establish associations. (Table 2). Those living in rooms/flats were less likely than those living in a whole house to report psychotic symptoms, and those earning an income were more likely to report psychotic symptoms compared to those who were not. Those reporting severe lack of social support (compared to a moderate lack), experiencing more than two recent life events, presence of CMD (a CIS-R score 12 or above) and past-year cannabis use were more likely to report psychotic symptoms.

Factors significant at the bivariate level (accommodation type, income, social support, life events, CIS-R score and past year cannabis use), as well as all factors significantly associated with area (household status, ethnicity, housing tenure, employment status excluding education due to small cell sizes) were entered forward stepwise into the logistic regression model. Recent life events, CMD and past year cannabis use remained independently associated with psychotic symptoms.

## 4. Discussion

This is the first study to our knowledge to explore associations with household status, ethnicity, housing tenure, accommodation type and social support, and also the first to compare rates of psychotic symptoms in two urban areas of differing levels of poverty in sub Saharan Africa. The study found that the prevalence of past-year psychotic symptoms (endorsing at least one PSQ item) in two areas of urban Dar es Salaam, Tanzania, was 3.9%. The rate was significantly higher in the poorer area Saba Saba (6.0%) compared to the relatively middle-income area of Ilala (2.1%) although this difference was no longer significant after adjustment for other factors. Factors independently associated with psychotic symptoms were two or more recent life events, presence of CMD and past-year cannabis use.

The annual psychotic symptom rate of 3.9% is consistent with findings from earlier Ethiopian studies. A prevalence of 6.0% was observed for psychotic symptoms using the Self-Reporting Questionnaire in rural Ethiopia [34], while rates of disorder were unsurprisingly lower with past month combined schizophrenia and schizoaffective disorder according to the CIDI 0.7% in a population-based urban sample [3], and psychotic illness 0.3% based on psychiatric interview [35]. A recent survey in Mozambique used key informants (the first person found in the randomly selected household able to answer on behalf of others) to identify disordered behaviour via vignette. The authors found higher lifetime prevalence of psychoses (4.4%) in the poorer rural area compared to 1.6% in Maputo city [4]. Using a similar methodology in Zanzibar, the rate of chronic psychosis was found to be 2.6/1000 and acute psychotic episodes 0.6/1000 [36]. Although population based surveys in the United States the Netherlands [37], and New Zealand [38], have found somewhat higher prevalence rates for psychotic symptoms (28%, 17.5% and 20.1% respectively), in Britain where the PSQ was used, the prevalence of psychotic symptoms was 5.5% [7], a figure comparable to the current results.

Psychotic symptoms were more prevalent in the more densely populated and relatively poorer Saba Saba than in Ilala. Compared to living in Ilala, living in Saba Saba was associated with unemployment, younger school leaving age and reporting more than one stressful life event in the six months preceding interview. Differences in these markers of poverty are consistent with the significant difference in estimated household monthly income between Saba Saba (17,751.61 TZS) and Ilala (22,307.58 TZS) [39] A higher prevalence of psychotic disorder was found in the poorer rural area compared to more affluent urban area in Mozambique [4].

Several limitations should be noted. While the sampling frame was well defined and based on the AMMP census from the preceding year, adequate supervision of the implementation of the survey was difficult for logistical reasons. The resulting missing data included a failure to record how often the person randomly selected for interview had moved since the last census round and therefore replaced by a new resident. The PSQ was not originally designed for sub-Saharan Africa and so was carefully scrutinised by local clinicians for content validity and put through a thorough process of translation and back translation but was not tested against a gold standard interview. As the prevalence of psychotic symptoms in general population samples is generally low, the overall sample size was not large enough to yield a large number of people reporting past-year psychotic symptoms, and therefore the power to detect associations was therefore limited. It prevented comparison of the two areas in relation to psychotic symptoms, and also prevented the possibility of comparing individuals of differing severity of psychotic symptoms against each other in relation to socio-demographic variables. We did not confirm probable psychosis with a follow up clinical interview by trained psychiatrists due to the high opportunity cost of such an exercise in a low income country with few psychiatrists. As always, the potential for measurement error when using screening instruments should be acknowledged given self-reported experiences may be subject to recall or social desirability response bias. Finally, the current findings are specific to the two wards in urban Dar es Salaam and are not necessarily applicable to other parts of Tanzania, particularly rural areas.

There was a significant positive association between the number of stressful life events and prevalence of psychotic symptoms. Tafari and colleagues [34] found that people who had experienced six or more stressful life events in the past year were two times more likely obtain a high psychosis score in Ethiopia. In the current study it was about five times greater for those with two or more events. The relationship between psychosis and stressful life events is well established in Britain [7,40], and it has been suggested that life events have greater influence on mental disorder than does poverty *per se* in the developing country context [41].

The results of a recent review suggest cannabis use increases the risk of psychosis [42], and while the small numbers reported in the current study should be interpreted with caution, the association between cannabis use and psychotic symptoms warrants further investigation in sub Saharan Africa. The association between psychotic symptoms and CMD has been previously reported in the British national surveys [43].

This is the first study to investigate the effect of perceived social support and size of primary support group on rates of psychotic symptoms in sub-Saharan Africa. Those with a severe lack of social support had higher rates of psychotic symptoms, comparable to Britain [39,44]. In contrast to Britain however, as the primary support group increased in size, so too did rate of disorder. While neither of these associations was significant and therefore the unexpected relationship the result of confounding by other variables, further investigation is warranted.

Unlike other studies, age, gender and marital status were not found to be significantly associated with psychotic symptoms. However, consistent with the review of schizophrenia in developing countries [2], prevalence was highest among males, people aged 25–34 and single people. Higher rates of psychotic illness were associated with older age and male gender in Mozambique [4], older age and single marital status in the most recent Ethiopia study [3] and being divorced, separated or widowed previously in Ethiopia [34]. In Britain, divorced and separated people had higher rates of probable psychosis in both sexes [45]. It would therefore be generally advantageous if rates were age standardized if comparisons are to be made with other studies.

The current paper also investigated household status, housing type, education, income and ethnicity, given their previously reported associations with psychotic symptoms in Britain [31,44] but none of these relationships was significant after adjustment for other variables.

## 5. Conclusions

Psychotic symptoms are prevalent and there are social inequities in their distribution. While the relationships of psychotic symptoms with social inequities are broadly similar in size and direction to those found elsewhere, there are some intriguing exceptions which deserve further study, including accommodation type and income. Social and economic development efforts to address poverty and unemployment in urban Tanzania will need to take mental health issues including mental disorders such as psychosis into account, and mental health has already been included in the National Health Sector Strategic Plan.

## Figures and Tables

**Table 1.** Prevalence of psychotic symptoms in the preceding year as measured by the five domains of the Psychotic Screening Questionnaire in two urban areas of Tanzania.

	**Past 12 month prevalence**		

	**Total n = 899 (%)**	**Saba Saba n = 418 (%)**	**Ilala n = 481 (%)**
**One or more symptoms**	35 (3.9)	25 (6.0)	10 (2.1)
** Strange experiences**	19 (2.1)	12 (2.9)	7 (1.5)
** Hallucinations**	10 (1.1)	8 (1.9)	2 (0.4)
** Thought insertions**	10 (1.1)	7 (1.7)	3 (0.6)
** Paranoia**	7 (0.8)	5 (1.2)	2 (0.4)
** Mania**	3 (0.3)	3 (0.7)	0 (0.0)

**Table 2.** Prevalence and odds ratios for one or more psychotic symptoms in the preceding year.

	**Sample size**	**Number of cases**	**Prevalence (%)**	**Unadjusted odds ratio**	**p–value**	**Adjusted odds ratio**	**p–value**
**Area**							
Saba Saba	418	25	6.0	1.00			
Ilala	481	10	2.1	0.33 (0.16–0.70)			
**Gender**							
Male	393	20	5.1	1.00			
Female	506	15	3.0	0.57 (0.29–1.13)	0.106		
**Age**							
16–24	275	10	3.6	1.00			
25–34	308	14	4.5	1.26 (0.55–2.89)	0.582		
35+	316	11	3.5	0.96 (0.40–2.29)	0.919		
**Marital status**							
Married/cohabitating	495	13	3.4	1.00			
Single	327	14	4.6	1.35 (0.67–2.75)	0.405		
Widowed/divorced/separated	75	8	4.0	1.17 (0.33–4.10)	0.804		
**Relationship to household head**							
Head	359	12	3.3	1.00			
Spouse/cohabit	290	9	3.1	0.93 (0.38–2.23)	0.864		
Other	250	14	5.6	1.72 (0.78–3.77)	0.180		
**Ethnic group**							
Black African	834	31	3.7	1.00			
Other	63	3	4.8	1.30 (0.38–4.36)	0.676		
**Employment status**							
Working	300	12	4.0	1.00			
Unemployed	49	3	6.1	1.57 (0.43–5.76)	0.500		
Economically inactive	496	17	3.4	0.85 (0.40–1.81)	0.676		
**Housing tenure**							
Owns	403	19	4.7	1.00			
Rents	463	16	3.5	0.72 (0.37–1.43)	0.350		
Rent free	29	0	0.0	–	–		
**Type of accommodation**							
Whole house	386	22	5.7	1.00			
Rooms/flat/other	510	13	2.5	0.43 (0.22–0.87)	0.019		
**Age left full time education**							
13 or under/Never went	52	3	5.8	1.00			
14–16	337	6	1.8	0.30 (0.07–1.22)	0.093		
17 or 18	212	12	5.7	0.98 (0.27–3.61)	0.976		
19+ yrs	208	12	5.8	1.00 (0.27–3.68)	1.000		
Still at school	71	2	2.8	0.47 (0.08–2.94)	0.422		
**Income**							
Yes	354	20	5.6	1.00			
No	476	13	2.7	0.47 (0.23–0.96)	0.037		
**Perceived social support**							
Severe lack	173	13	7.5	1.00			
Moderate lack	288	6	2.1	0.26 (0.10–0.70)	0.008		
No lack	341	15	4.4	0.57 (0.26–1.22)	0.146		
**Size of primary social support group**							
0–3	130	5	4.0	1.00			
4 to 8	411	13	3.0	0.82 (0.29–2.34)	0.705		
9 or more	358	17	5.0	1.25 (0.45–3.45)	0.672		
**Number of life events**							
None	576	12	2.1	1.00		1.00	
1	206	7	3.4	1.65 (0.64–4.26)	0.298	1.36 (0.45–4.16)	0.588
2 or more	117	16	13.7	7.45 (3.42–16.21)	0.000	6.43 (2.58–16.02)	0.000
**CIS R**							
<12	872	30	3.4	1.00		1.00	
>12	27	5	18.5	6.38 (2.26–17.99)	0.000	3.33 (1.05–10.58)	0.042
**Hazardous alcohol use**							
No	848	31	3.7	1.00			
Yes	51	4	7.8	2.24 (0.76–6.62)	0.143		
**Past year cannabis**							
No	888	33	3.7	1.00		1.00	
Yes	7	2	28.6	10.36 (1.94–55.4)	0.006	8.23 (1.23–54.87)	0.030
